# Commonly expressed key driver genes in the pathogenesis of viral and non-viral liver cancer

**DOI:** 10.1371/journal.pone.0349647

**Published:** 2026-11-06

**Authors:** Melika Zamanian, Niloofar Bajool, Yasamin Haghir-Sharif-Zamini, Seyedeh-Sanaz Araban, Farbod Nassiri, Seyedeh-Nasim Mirbahari, Rayhaneh Ahangar-Parvin, Roshanak Shams, Massoud Vosough, Sara Taleahmad

**Affiliations:** 1 Department of Applied Cell Sciences, Faculty of Basic Sciences and Advanced Medical Technologies, Royan Institute, ACECR, Tehran, Iran; 2 Department of Stem Cells and Developmental Biology, Cell Science Research Center, Royan Institute for Stem Cell Biology and Technology, ACECR, Tehran, Iran; 3 Department of Cell and Developmental Biology, Faculty of Basic Sciences and Advanced Technologies in Medicine, University of Science and Culture, Tehran, Iran; 4 Department of Regenerative Medicine, Cell Science Research Center, Royan Institute for Stem Cell Biology and Technology, ACECR, Tehran, Iran; 5 Department of Tissue Engineering, Faculty of Basic Sciences and Advanced Technologies in Medicine, Royan Institute, ACECR, Tehran, Iran; 6 Department of Cell Engineering, Cell Science Research Center, Royan Institute for Stem Cell Biology and Technology, ACECR, Tehran, Iran; 7 Bone and Joint Reconstruction Research Center, Department of Orthopedics, School of Medicine, Iran University of Medical Sciences, Tehran, Iran; 8 Experimental Cancer Medicine, Institution for Laboratory Medicine, Karolinska Institute, Stockholm, Sweden; The First Hospital of Jilin University, CHINA

## Abstract

**Background:**

Hepatocellular carcinoma (HCC) is a common and lethal liver cancer primarily associated with viral infections like hepatitis B virus (HBV) and hepatitis C virus (HCV), as well as non-viral causes. Understanding the molecular mechanisms of these subtypes is essential for identifying potential biomarkers and therapeutic targets.

**Methods:**

In this study, an integrated bioinformatics approach was used to analyze publicly available datasets related to HBV-associated, HCV-associated, and non-viral HCC. Functional enrichment analyses and protein–protein interaction (PPI) networks were constructed to identify key hub genes.

**Results:**

Our analysis identified dysregulated genes across all HCC subtypes. Among these, *GPC3* and *SPARCL1* were significantly upregulated, while *ALB* and *HNF4α* were downregulated, and are involved in critical cancer pathways such as Wnt, PI3K/Akt, and p53 signaling. Functional analysis linked these DEGs to essential processes in tumorigenesis, including cell cycle regulation and immune modulation. Notably, *SPARCL1* has been identified as a potential inhibitor of *SPP1*, a key driver of HCC. Validation using databases such as GEPIA and TNMplot confirmed the dysregulation of these genes in numerous HCC tissue samples and their significant association with patient prognosis.

**Conclusion:**

This study provides a comprehensive overview of HCC pathogenesis by suggesting a shared gene profile between viral and non-viral forms, paving the way for universal diagnostic markers and targeted therapies while highlighting the need for further experimental validation to advance precision medicine in HCC.

## 1 Introduction

Liver cancer is a significant public health problem worldwide, ranking as the sixth most common cancer and the third leading cause of cancer-related deaths [1]. Viral infection, non-alcoholic fatty liver disease (NAFLD), and alcohol abuse are well-recognized as the three primary risk factors associated with the development of liver cancer [2]. Hepatocellular carcinoma (HCC) and intrahepatic cholangiocarcinoma (ICC) are the two most common primary liver cancers. HCC arises from the main parenchymal cells of the liver, hepatocytes, whereas ICC arises from biliary ducts. HCC accounts for almost 80% of all primary liver cancer cases worldwide [3]. Infection with hepatitis B (HBV) or C (HCV) viruses is a major risk factor for HCC. Globally, approximately 80% of virus-associated HCC cases are attributed to HBV infection [4]. HBV belongs to the Hepadnaviridae family and has partially double-stranded DNA, whereas HCV is a member of the Flaviviridae family and has a single-stranded RNA genome [5,6]. Several signaling pathways, including Wnt/β-catenin, TGF-β, MAP kinase, JAK/STAT, p53, epidermal growth factor, and stress response, have been linked to HCC development [7–9].

It has been reported that chronic hepatitis B (HBV) and chronic hepatitis C (HCV) patients have different gene expression profiles in their livers. Pro-apoptotic and DNA repair responses were predominant in HBV, whereas inflammatory and anti-apoptotic pathways were more prevalent in HCV [10,11]. Several immune response- and cytoskeleton-related genes, such as actin, tubulin, and vimentin, were upregulated in HCV-related HCC. In contrast, collagen IV and matrix metalloproteinases were upregulated in HBV-related HCC. Chronic inflammation, oxidative damage, and repair responses contribute to the development of liver fibrosis and cirrhosis. Liver fibrosis, cirrhosis, and HCC over a prolonged period are likely caused by processes that modulate cellular immunity and metabolism. Finally, some HCV proteins alter cell proliferation and checkpoint mechanisms during the cell cycle [12]. Bioinformatics is a computational approach to biological data analysis that aids in the organization and integration of data from multiple sources. This method develops and applies models and algorithms to predict the behavior and response of cancer cells and tissues, optimize experiments, and improve accuracy. The application of bioinformatics in cancer research has significantly enhanced our understanding of complex data, facilitated the integration of fundamental and clinical research, and successfully uncovered and confirmed innovative biomarkers and targets for HCC [13,14]. Moreover, *in silico* studies have been beneficial to HCC researchers because they have helped identify key genes, pathways, and signaling networks involved in therapeutic interventions. This study used comprehensive bioinformatics analysis to identify molecular targets that contribute to HCC development caused by HBV, HCV, or other risk factors. The aim was to identify specific molecular pathways and targets involved in HCC development that could be used as therapeutic targets for treatment or as biomarkers for diagnosis. The procedure of this study is illustrated as a flowchart in Fig. 1.

**Fig 1:**
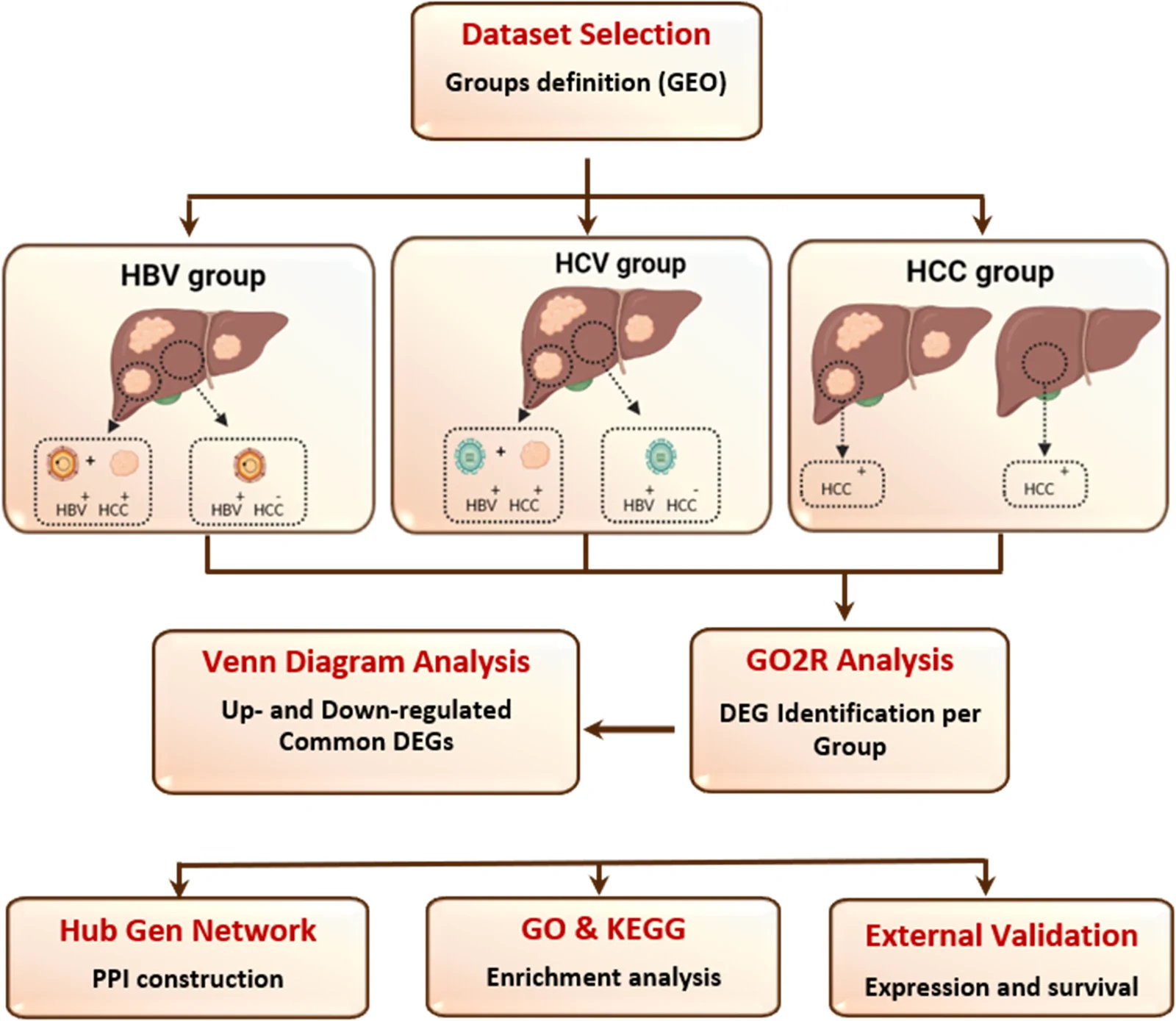
Schematic workflow of the bioinformatics analysis performed in this study. The workflow includes dataset selection, differential expression analysis, identification of common differentially expressed genes, functional enrichment analysis, protein–protein interaction network analysis, hub gene identification, and external validation.

## 2 Methods

### 2.1 Data collection

This study employed a top-down approach. The procedure for identifying and validating a confident set of differentially expressed genes (DEGs) in three distinct groups was as follows: the HBV group consisted of HBV-positive patients with HCC versus patients without HCC as a control; the HCV group used the same logic (referred to HCV-positive patients with HCC versus without HCC as a control), comparing HCV-infected patients with or without HCC (control); and the HCC group consisted of HCC patients and healthy individuals who did not have HCC or hepatitis.

The gene expression datasets were obtained from the Gene Expression Omnibus microarray database (GEO; https://www.ncbi.nlm.nih.gov/geo). Table 1 presents the GEO accession numbers of the selected studies. From each selected GEO dataset, HBV-positive hepatocellular carcinoma (HCC) tissue samples were included and compared against their corresponding HBV-negative or adjacent non-tumor control samples. All available samples meeting this criterion within each dataset were included in the analysis, and no additional sample-level exclusions were applied.

**Table 1:** Defines groups, inclusion and exclusion criteria.

	GSE	Platform name	Source of sample	Number of samples	DEGs	Up	Down
**HBV**	GSE19665	GPL570 [HG-U133_Plus_2] Affymetrix Human Genome U133 Plus 2.0 Array	Tumor and non-tumor tissues from HBV-infected liver	Tumor: 5 Non-tumor: 5	586	136	450
	GSE121248	GPL570 [HG-U133_Plus_2] Affymetrix Human Genome U133 Plus 2.0 Array	Tumor and non-tumor tissues from HBV-infected liver	Tumor: 70 Non-tumor: 37	4985	2522	2463
**HCV**	GSE44074	GPL13536 Kanazawa Univ. Human Liver chip 10k	Tumor and non-tumor tissues from HCV-infected liver	Tumor: 17 Non-tumor: 35	280	120	160
	GSE17856	GPL6480 Agilent-014850 Whole Human Genome Microarray 4x44K G4112F (Probe Name version)	Tumor and non-tumor tissues from HCV-infected liver	Tumor: 49 Non-tumor: 44	92	7	85
**HCC**	GSE62232	GPL570 [HG-U133_Plus_2] Affymetrix Human Genome U133 Plus 2.0 Array	Tumor and non-tumor liver	Tumor: 81 Non-tumor: 10	7209	2266	4943
	GSE45050	GPL6244 [HuGene-1_0-st] Affymetrix Human Gene 1.0 ST Array [transcript (gene) version]	Tumor and non-tumor liver	Tumor: 6 Non-tumor: 3	141	40	101

### 2.2 Identification of differentially expressed genes (DEGs)

Differential expression gene analysis was performed using the GEO2R online tool, which compared the case and control samples. Statistically significant DEGs were filtered using mathematically determined cut-off criteria as follows: adjusted p-value < 0.05, and logFC ± 0.56 (Table 1 and S1 Table). Similar upregulated and downregulated DEGs among the HBV, HCV, and HCC groups were identified using Venn diagrams.

### 2.3 Functional enrichment analysis

To investigate the biological significance of DEGs, Gene Ontology (GO) and Kyoto Encyclopedia of Genes and Genomes (KEGG) pathway enrichment analyses of up- and downregulated genes were performed using the Database for Annotation Visualization and Integrated Discovery (https://david.ncifcrf.gov) and Enrichr (https://maayanlab.cloud/Enrichr/). P-values less than 0.05 were considered the cut-off criterion.

### 2.4 Protein-protein interaction (PPI) network construction

To investigate the interactions between the up- and downregulated DEGs, the STRING database was used. PPI network analysis was performed using Cytoscape (v3.9.1) with the CytoHubba plugin. The top 20 proteins were ranked based on degree, betweenness, eccentricity, and closeness criteria. Finally, Venn diagrams were used to identify hub genes common to these four criteria. The selected hub gene network was visualized using the Gephi (v0.9.2) database.

### 2.5 Hub gene identification

The GEPIA database was used to verify the hub genes in (LIHC) development, utilizing data from the TCGA and GTEx projects [15]. Candidate genes were uploaded to the GEPIA database, and logFC and p-values were used as cut-off criteria. The Kaplan–Meier plot was used to indicate the overall survival of the candidate genes in patients with HCC based on the TCGA-LIHC cohort, according to their expression in the GEPIA database. Patients were stratified into high- and low-expression groups using the median expression value (50% cutoff). This validation was also performed using the TNMplot database to analyze gene and protein expression in a larger sample size.

## 3 Results

### 3.1 Enrichment analysis of DEGs

Using GEO2R analysis, 586 and 4985, 280 and 92, 7209 and 141 DEGs were identified in the HBV, HCV, and HCC groups, respectively (Table 1). Venn diagrams of upregulated and downregulated genes among the three groups (HBV, HCV, and HCC) showed that 33 and 113 genes were common among all three groups, respectively (Fig 2A). According to GO analysis, our results indicated that cell cycle, focal adhesion, p53 signaling pathway, ECM-receptor interaction, and hepatocellular carcinoma were the most significant KEGG pathways related to upregulated genes that were common among the three groups (Fig 2A). In addition, KEGG pathway analysis of overlapping downregulated genes in the three groups revealed association with various pathways, including drug metabolism, chemical carcinogenesis, complement and coagulation cascades, glycolysis/gluconeogenesis, PPAR signaling pathway, TGF-beta signaling pathway, AMPK signaling pathway, and FoxO signaling pathway (Fig 2A).

**Fig 2:**
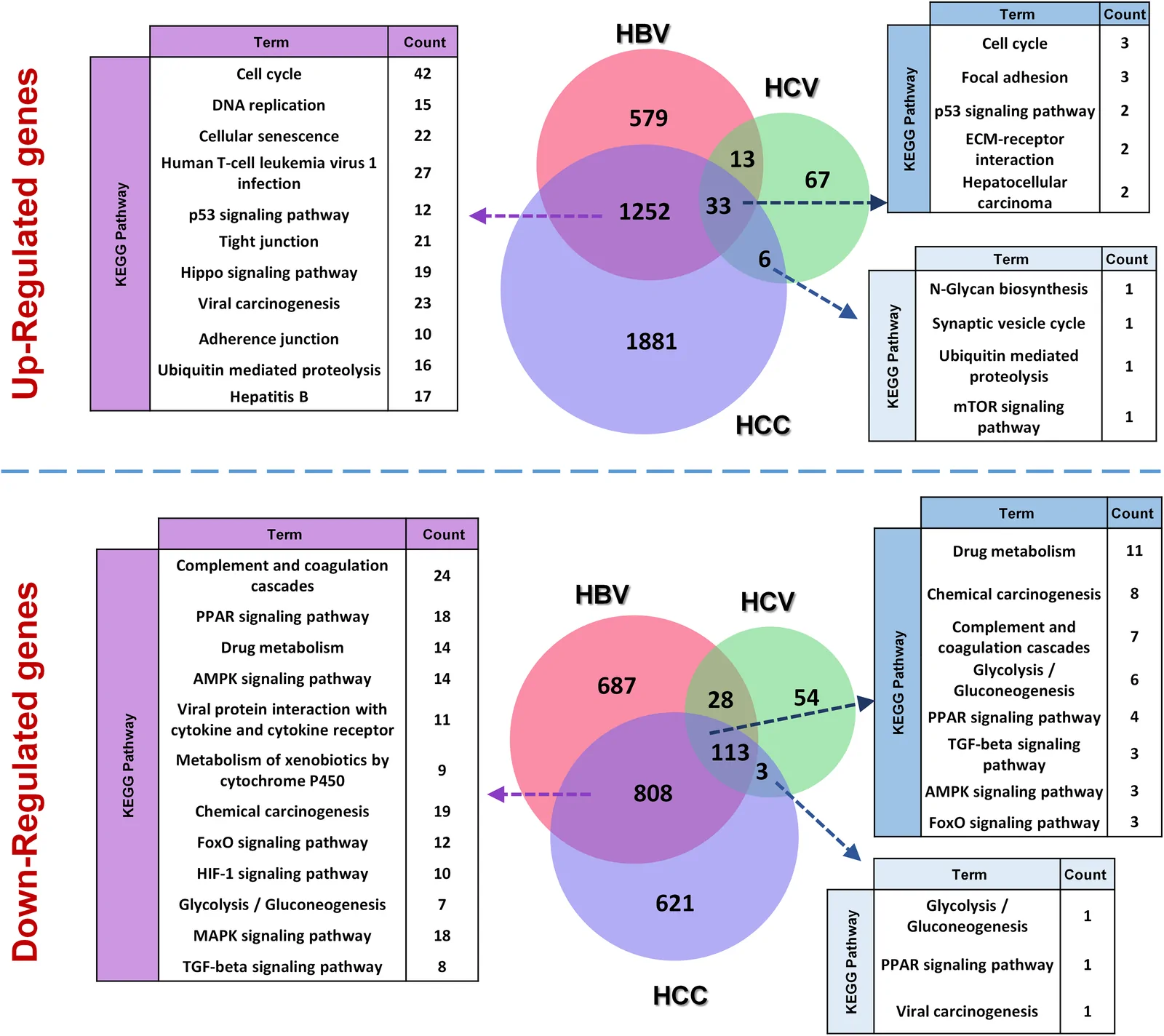
Enrichment analysis of upregulated and downregulated DEGs. KEGG analysis between the HBV, HCV, and HCC groups.

KEGG pathway analysis of genes in HBV-HCC identified the following: cell cycle, DNA replication, cellular senescence, human T-cell leukemia virus 1 infection, p53 signaling pathway, tight junction, Hippo signaling pathway, viral carcinogenesis, adherence junction, ubiquitin-mediated proteolysis, and hepatitis B for upregulated genes and complement and coagulation cascades, PPAR signaling pathway, drug metabolism, AMPK signaling pathway, viral protein interaction with cytokine and cytokine receptor, metabolism of xenobiotics by cytochrome P450, chemical carcinogenesis, FoxO signaling pathway, HIF-1 signaling pathway, Glycolysis/Gluconeogenesis, MAPK signaling pathway, and TGF-beta signaling pathway for downregulated genes.

Additionally, KEGG signaling pathway analysis for upregulated and downregulated genes between HCV and HCC groups indicated that upregulated genes are involved in N-glycan biosynthesis, synaptic vesicle cycle, ubiquitin-mediated proteolysis, and mTOR signaling pathway, and downregulated genes are associated with Glycolysis/Gluconeogenesis, PPAR signaling pathway, and viral carcinogenesis pathways.

GO analysis in the Enrichr database identified gene functions in the defined groups. The top ten BP, MF, and CC were selected based on p-value < 0.05 (S1 Fig). The results showed that the most significant BP, CC, and MF for upregulated genes in the HBV-HCV-HCC group were involved in regulation of positive regulation of cellular metabolic process, Vacuolar lumen, and Signal sequence binding, respectively and for the downregulated genes, cellular zinc ion homeostasis, collagen-containing extracellular matrix and zinc ion binding were the most significant BP, CC and MF, respectively (S1 Fig).

For the HBV-HCC group, the BP, CC, and MF results of upregulated genes revealed that these genes were mainly associated with microtubule cytoskeleton organization, nucleus, and RNA binding, respectively. Downregulated genes were particularly enriched in cellular amino acid catabolic processes, intracellular organelle lumen, and oxidoreductase activity for BP, CC, and MF, respectively (S1 Fig). In addition, these three aspects for the HCV-HCC group were SRP-dependent co-translational proteins targeting the membrane, lytic vacuole membrane, P-type proton-exporting transporter activity, and regulation of intracellular cholesterol transport, microbody lumen, and acetyl-CoA C-acyltransferase activity for upregulated genes and downregulated genes, respectively (S1 Fig).

This analysis identified specific genes for each group (S2 Fig). A Venn diagram represents 579 upregulated and 686 downregulated genes specific to HBV. These genes were associated with viral carcinogenesis and ECM-receptor interaction pathways for upregulated genes, and complement and coagulation cascades, metabolic pathways, chemical carcinogenesis, and drug metabolism for downregulated genes. In the context of the HCV group-specific genes, most of them were downregulated, related to the steroid biosynthesis pathway, and upregulated genes involved in fructose and mannose metabolism. In the HCC group, 1881 upregulated and 621 downregulated genes had specific expressions that did not exist in the HBV and HCV groups. The phosphatidylinositol signaling system, AMPK signaling pathway, mTOR signaling pathway, Notch signaling pathway, Hippo signaling pathway, PD-L1 expression, and PD-1 checkpoint pathway in cancer were top-enriched pathways for upregulated genes, and biosynthesis of cofactors, metabolic pathway, and tight junctions were related to downregulated genes.

### 3.2 Analysis of protein-protein interactions (PPI)

To construct the PPI network, common upregulated and downregulated genes among the three groups were analyzed using the STRING online database and Cytoscape software. The Cytoscape CytoHubba plugin was then used to identify the top 20 hub genes based on criteria such as betweenness, closeness, degree, and EcCentricity. A Venn diagram was used to the identify genes that met all four criteria. As shown in S3A Fig, 15 genes were identified as upregulated (*CCNB1*, *H2AFZ*, *LMNA*, *KPNA2*, *ACLY*, *VWF*, *SPARCL1*, *MCM3*, *ANXA2*, *GPC3*, *TXNRD1*, *PLOD3*, *KRTCAP2*, *COL15A1*, *FDPS*) and 6 genes were identified as downregulated (*ALB*, *GPT*, *KLKB1*, *CYP2B6*, *ADH4*, *PLG*). The relationships between these upregulated and downregulated genes were visualized using Gephi, as shown in S3B Fig. The relationships between these upregulated and downregulated genes were visualized using Gephi, as shown in S2B Fig.

### 3.3 Identifying the function of hub genes

Based on the literature, the identified upregulated and downregulated genes in HCC were further examined for their functions. The upregulated genes were classified into three categories based on their functions: proliferation, metastasis, and other cancer-related contexts. Notably, *GPC3* and *SPARCL1* exhibited logFC values greater than 1 in all three groups (Table 2).

**Table 2:** LogFC of up- and downregulated hub genes in comparison with GEPIA results.

Gene name	LogFC in the HBV group	LogFC in the HCV group	LogFC in the HCC group	LogFC in GEPIA
***MCM3***	1.07	0.78	1.99	1.9
***H2AFZ***	0.78	0.68	1.5	1.6
***CCNB1***	2.14	0.9	2.88	4
***GPC3***	2.68	2.95	3.42	7.3
***SPARCL1***	1.16	1.03	1.77	1.97
***VWF***	0.94	0.93	1.64	3
***ANXA2***	1.16	0.62	2.49	2
***COL15A1***	2.97	0.72	3.99	4.15
***PLOD3***	0.7	0.74	1.38	1.37
***TXNRD1***	1.72	0.68	1.44	1.4
***ACLY***	0.76	0.89	1.56	1.35
***KPNA2***	1.33	0.83	1.54	2.17
***FDPS***	0.86	1.04	1.74	1.42
***KRTCAP2***	0.8	1.04	1.17	1.74
***ALB***	−0.88	−1.21	−1.3	−0.56
***GPT (ALT1)***	−0.68	−0.98	−1.33	−0.98
***KLKB1***	−1.68	−1.05	−3.14	−0.98
***CYP2B6***	−2.38	−0.68	3.47	−2.32
***ADH4***	−1.21	−1.89	−3.01	−1.83
***PLG***	−1.17	−1.16	−2.44	−0.74

### 3.4 Validation of candidate genes in GEPIA, TNMplot database

By examining the expression of candidate genes associated with HCC: *GPC3, SPARCL1, THBS4, CTNNB1, VWF,* and *ADAMTS13* in LIHC relative to normal liver tissue in the TCGA data, we found a significant upregulation of *GPC3, SPARCL1, LEF1, THBS4,* and *VWF* and downregulation of *ADAMTS13*. Analysis of the protein and gene expression of these genes in TNMplot is also in line with our data. Kaplan-Meier analysis showed a correlation between the expression levels of *GPC3, SPARCL1, THBS4, LEF1, VWF,* and *ADAMTS13* (high and low), the overall survival rate of patients in 120 months, in a population of 182 people in both groups, Fig 3. In this plot, the blue line is related to the survival rate of individuals with low gene expression, while the red line indicates the survival rate of those with high gene expression. Moreover, the box plot illustrates the gene expression levels in normal and cancerous tissues, with red color representing cancerous tissue and blue one using for normal tissue. Based on the log-rank, the differences between the two curves for all of these genes are statistically significant. Additionally, the validation of these candidate genes was conducted using the TNMplot database (S4 Fig).

**Fig 3:**
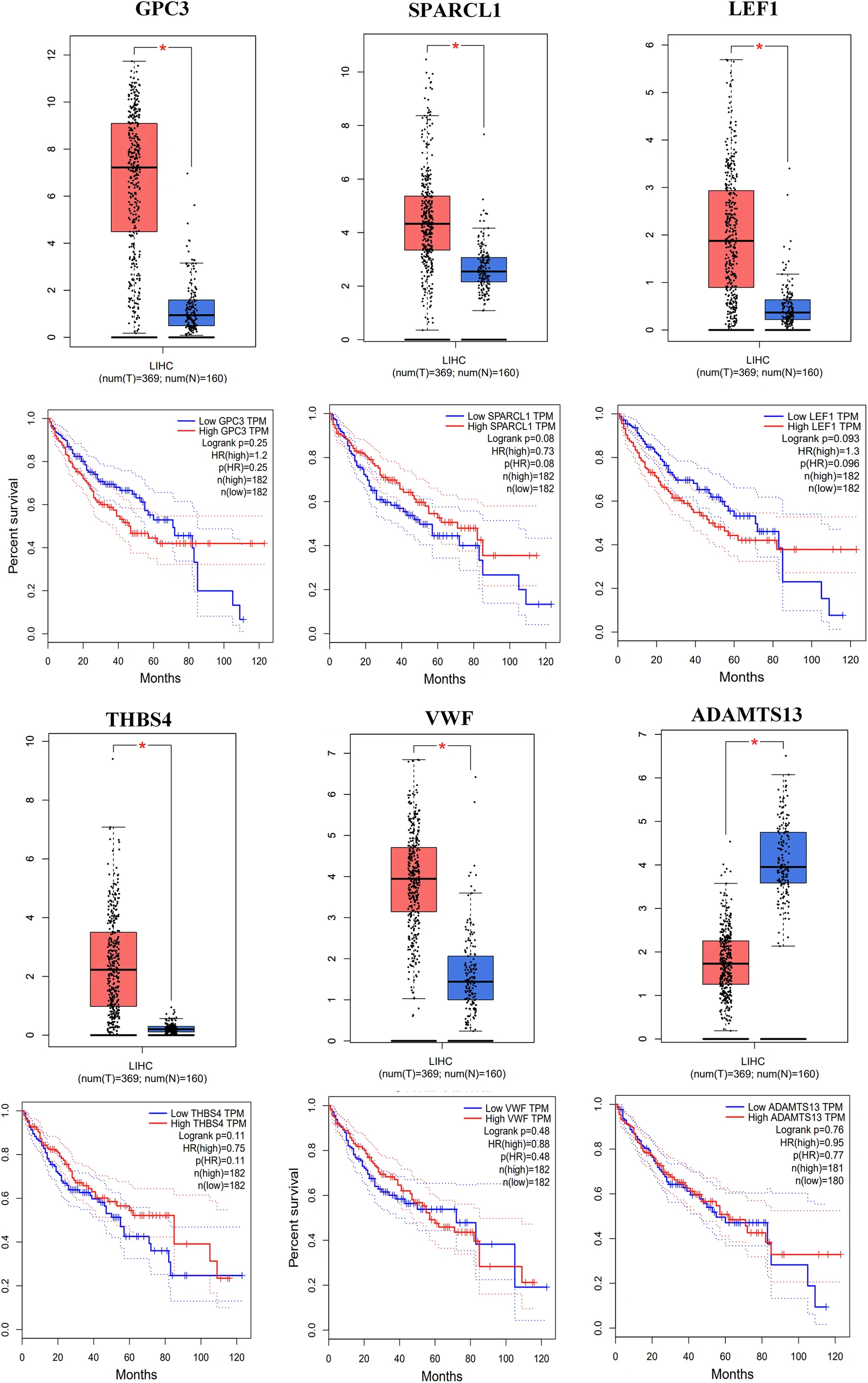
GEPIA analysis for important genes (*GPC3*, *SPARCL1*, *LEF1*, *THBS4*, *ADAMTS13*, and *VWF*) associated with hepatocellular carcinoma. In a survival plot, the blue line indicates the individuals with low gene expression, while the red line indicates those with high gene expression. In the box plot, red indicates cancerous tissue, and blue indicates normal tissue.

## 4 Discussion

Nowadays, there is no specific and effective treatment for individuals diagnosed with HCC. Furthermore, the absence of early clinical symptoms results in only 40% of HCC cases being identified at an early stage [16–18]. Therefore, identifying molecular targets in HCC patients, irrespective of their risk factors, could greatly assist in early detection and targeted treatment. In this study, we conducted an analysis of microarray data from various studies on HCC and its associated risk factors, including hepatitis B virus (HBV) and hepatitis C virus (HCV) infections, as presented in Table 1. A comparative analysis of these three groups revealed 15 upregulated hub genes and six downregulated ones across all three groups. Table 2 shows that *GPC3* and *SPARCL1* are among the upregulated hub genes, with log-fold changes greater than 1.

Glypican-3 (GPC3), as an upregulated gene in this study, is an oncofetal protein that may serve as a viable therapeutic target for HCC treatment. Among the signaling pathways associated with the identified hub genes, the Wnt signaling pathway is particularly relevant due to the established role of GPC3 in regulating Wnt/β-catenin signaling during HCC development. Additionally, GPC3 has been identified as a modulator of various signaling pathways in the liver, including the Wnt, YAP, and hedgehog pathways, all of which play significant roles in liver cancer pathogenesis [19]. Our research uncovered some mechanisms related to the GPC3 protein that are shown in Fig 4B. GPC3 acts as a co-receptor for Wnt proteins and engages in interactions via its cysteine-rich hydrophobic groove located in the N-lobe [19,20]. Capurro et al. showed that overexpression of GPC3 promotes proliferation and growth in HCC cell lines by enhancing activation of the Wnt canonical signaling pathway [21]. The activation of the Wnt signaling pathway leads to the translocation of β-catenin into the nucleus, where in interact with TCF/LEF proteins to form a complex. This process triggers the transcription of target genes involved in cell proliferation, cell cycle progression, metastasis, and invasion [22]. Overexpression of LEF1 is associated with the progression of several cancers, and it is closely related to the poor prognosis of HCC [23,24]. Therefore, the upregulation of GPC3 and the involvement of Wnt-related components observed in our study suggest that activation of the GPC3/Wnt/β-catenin axis may contribute to HCC progression through regulation of proliferation, invasion, and metastatic processes. Another biologically relevant pathway associated with the dysregulated genes identified in this study is the PI3K/Akt signaling pathway, which regulates diverse cellular processes, including cell growth, proliferation, apoptosis, metabolism, and angiogenesis. Thrombospondin-4 (THBS4), one of the upregulated genes in this study, is involved in the PI3/Akt pathway. THBS4 is a calcium-binding glycoprotein secreted as a globular pentameric complex into the extracellular matrix. It is one of the main developmental factors in embryogenesis [25]. This protein is involved in various crucial cellular processes. Wu, H. et al., discovered that THBS4 has shown higher expression in 40.47% of HCC tissues compared to adjacent non-cancerous tissues. They suggested that THBS4 is potentially involved in HCC pathogenesis [26]. In addition, another study indicated that THBS4 overexpression led to the proliferation and metastasis of HCC cells by interacting with integrin β1 (ITGB1), and modulating the FAK/PI3K/AKT pathway in both HCC patients’ tissues and cell lines. As we found increased expression of ITGB1 and THBS4 (Fig 4B), it can be suggested that their interaction leads to HCC pathogenesis in HBV and non-viral HCC group of study [27]. Previous studies demonstrated that the activity of PI3K and Akt can trigger the action of downstream transcription factor proteins, such as forkhead box O protein (FOXO) [28,29]. FOXO directly interacts with genes or other transcription factors to regulate gene transcription. An imbalance in *FOXO1* expression has been detected in various cancers. This irregularity impacts a range of cellular functions, such as apoptosis, cell cycle control, DNA damage repair, carcinogenesis, glucose metabolism, and tumor immunity [30]. In HCC, the Akt signaling pathway suppresses *FOXO1* transcriptional activity. Consequently, the inhibition of *FOXO1* results in heightened epithelial-mesenchymal transition (EMT), enhancing the migratory and invasive characteristics of HCC cells [31]. In this study significantly up-regulation of *FOXO1* (Fig 4B) among HBV and HCC groups was linked to HCC progression, which could contribute to HCC progression in combination with other altered pathway components.

**Fig 4:**
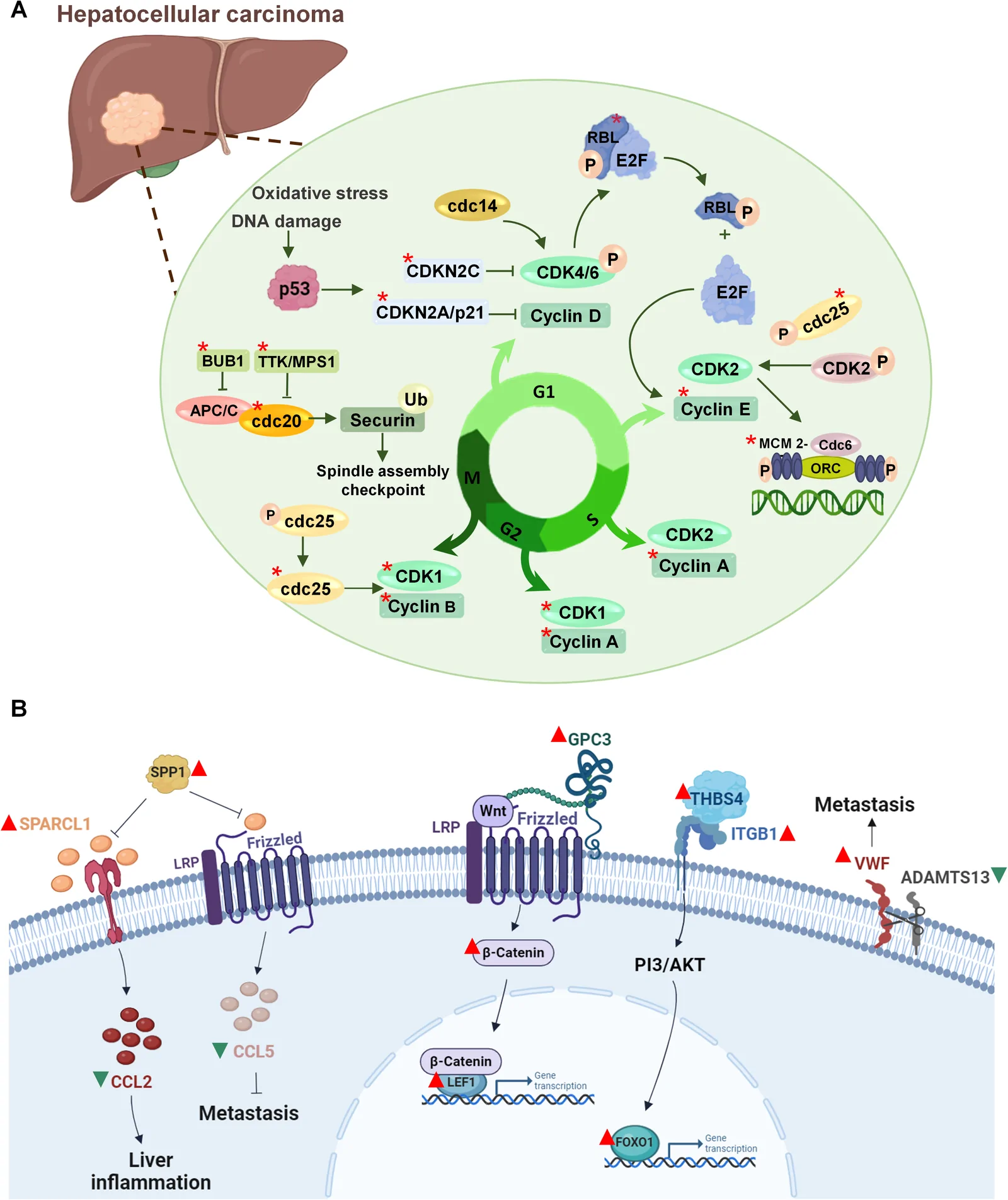
Schematic representation of signaling pathways and molecular interactions associated with hepatocellular carcinoma. **(A)** Commonly dysregulated genes involved in cell-cycle regulation identified across the HBV, HCV, and HCC groups. **(B)** Schematic representation of selected signaling mechanisms involving GPC3, SPARCL1, THBS4, VWF, ADAMTS13, and related downstream molecules implicated in HCC progression. Upward and downward arrows indicate genes with increased and decreased expression, respectively. The authors created the schematic based on the results of the present study and published literature.

The p53 signaling pathway emerged as another notably enriched route recognized through KEGG analysis. In our research, CCNB1 was recognized as an elevated hub gene linked to this pathway. The enhancement of the p53 signaling pathway alongside the overexpression of CCNB1 may suggest modified cell cycle regulation in the examined HCC cohorts. This discovery further substantiates the possible role of dysregulated cell cycle-associated signaling in the advancement of HCC.

Given the enrichment of the cell cycle pathway across the three study groups, we further investigated the roles of several upregulated cell cycle-related proteins in HCC progression (S2 Table). Several proteins associated with the spindle assembly checkpoint (SAC), which are often overexpressed in human cancers, include BUBs, MADs, and threonine tyrosine kinase (TTK), as shown in Fig 4A [32]. TTK overexpression results in the expansion of centrosomes, overstimulation of the spindle assembly checkpoint (SAC), and instability of chromosomes. This contributes to the development of tumors and has a notable impact on HCC [33]. A study showed that the levels of TTK protein are noticeably higher in cancerous liver tissue samples from patients with liver cancer than in adjacent healthy liver tissues. When TTK is silenced in vivo with TTK siRNAs, it restricts the spread of liver cancer cells within the liver. Therefore, they suggested that specifically inhibiting TTK could be beneficial as a supplementary treatment in managing liver cancer [34]. *In vitro* and *in vivo* studies showed that up-regulation of BUB1B correlated with HCC progression by enhancing the proliferation, migration, invasion, epithelial‐mesenchymal transition (EMT), and metastases of HCC cell lines [35].

Von Willebrand factor (VWF) is another hub gene that showed up-regulation in all three groups of this study. VWF is a glycoprotein that plays a crucial role in the adhesion of platelets along the endothelium and is a risk factor for venous thrombosis [36]. Its remarkable adhesive properties imply how tumor cells or leukocytes can adhere to the vascular endothelium. VWF can facilitate tumor metastasis through increasing extravasation of tumor cells by activating pro-inflammatory signaling and elevating vascular permeability, supporting adherence of tumor cells to endothelial cells, and stimulating angiogenesis in the tumor microenvironment [37,38]. The results of this study showed that ADAMTS13 was downregulated. This enzyme degrades VWF and regulates its size and function. This enzyme family, as a metalloproteinase, may influence angiogenesis, potentially contributing to cancer development. ADAMTS13 deficiency leads to a decrease in VWF breakdown, causing UL-VWF multimer accumulation [39,40]. Takaya et al. suggested that the ratio of VWF/ADAMTS13 could be a novel biomarker for early diagnosis of HCC in cirrhotic patients [41]. So, a dysregulated balance of the ratio between VWF and ADAMTS13 can be one of the reasons for HCC incidence between all three groups in this study (Fig 4B).

The other important hub gene increased in this study is *SPARCL1*. Several studies have shown its down-regulation in numerous cases of human epithelial cancer, suggesting its potential function as a tumor suppressor [42–45]. A study conducted by Zhao et al. provided evidence that SPARCL1 induces WNT/β-catenin signaling activation through interaction with frizzled and LRP receptors, which leads to stabilization of the WNT-receptor complex. Activation of WNT/β-catenin signaling is implicated in the inhibitory mechanisms exerted by SPARCL1 in osteosarcoma (OS) metastasis. Moreover, the paracrine effect of SPARCL1 on macrophage recruitment is mediated by chemokine ligand 5 (CLL5) secretion from OS cells, which results from activation of Wnt/β-catenin signaling [46]. Furthermore, Lau et al. have shown a positive correlation between SPARCL1 up-regulation and HCC progression as well as angiogenesis [47]. Through Sparcl1 protein binding to Toll-like receptor 4 (TLR4), the production of C-C motif chemokine ligand 2 (CCL2) in hepatocytes is promoted, and hepatic inflammatory responses in the mouse liver are triggered. CCL2 is a chemokine that acts as a chemoattractant for leukocyte trafficking, growth, and activation at the inflammatory sites. Consequently, there is an induction of hepatic macrophage recruitment and subsequent liver inflammation, thereby facilitating the non-alcoholic steatohepatitis (NASH) progression. Therefore, *SPARCL1* suppression may serve as a potential therapeutic approach for NASH treatment [48,49]. Our study showed SPARCL1 up-regulation, and as its downstream proteins, CCL2 and CCL5, demonstrated down-regulation, therefore SPARCL1 activity might be inhibitory in this case. According to Zhang et al., SPP1 (secreted phosphoprotein 1) has been suggested to neutralize the inhibitory effect of SPARCL on the proliferation, invasion, and migration of HELA cells [42]. The critical role of SPP1 in HCC initiation, progression, and metastasis has been elucidated by several studies. Sun et al. propose that SPP1 holds promise as a novel target for the prevention, diagnosis, and therapeutic treatment of HCC [50]. In addition, one study reported its overexpression in HBV-related metastatic HCC [51]. Plasma proteomic profiling of HCC patients with different etiologies and comparison to a control group revealed that OPN has greater sensitivity than AFP (Alpha-fetoprotein, a common biomarker for liver cancer) in HCC diagnosis in all HCC study groups [52]. In our study, both SPP1 and SPARCL1 were upregulated. Therefore, whereas earlier research has indicated a relationship between these proteins, our expression data do not explicitly corroborate an inhibitory influence of SPP1 on SPARCL1. Instead, their co-upregulation may indicate that both proteins are involved in the altered tumor microenvironment associated with HCC progression (Fig 4B).

Also, we find down-regulation of some important in HCC progression. Albumin (ALB), as one of the hub genes that is recognized, is a significant prognostic marker for HCC. Several studies have indicated that ALB can influence HCC cell proliferation. In addition, albumin level reduction is linked to the larger HCC tumors presence [53,54]. Fu et al. found that the inhibition of ALB can enhance the migration and invasion of HCC cells by elevating levels of MMP2 and MMP9 [55]. Another important downregulated gene is ADH4 (Alcohol dehydrogenase 4), which several studies correlate to this finding and recognize the importance of ADH4 in HCC progression [56,57]. Additionally, we observed down-regulation of hepatocyte nuclear factor 4α (HNF4α), which is linked to numerous liver-specific genes in several procedures such as metabolism, development, and differentiation of endoderm, and morphogenesis [58]. Several studies have indicated that HNF4α expression is altered in hepatocellular carcinoma (HCC) and plays a significant role in the onset and progression of the disease [59,60]. Identifying these genes and pathways associated with HCC, irrespective of its risk factors, can serve as biomarkers for early diagnosis, monitoring, and treatment of affected individuals. However, it is essential to validate these genes through further *in vitro* and *in vivo* studies.

### 4.1 Study limitations

This study is limited by its reliance on publicly available datasets and the lack of experimental validation. Within each etiology group (HBV-associated, HCV-associated, and non-viral HCC), multiple GEO datasets generated on different microarray platforms were pooled, and the resulting pooled etiology groups were subsequently compared with one another; this approach may introduce inter-dataset heterogeneity and potential batch effects, and no explicit batch-correction method was applied prior to pooling, which should be considered when interpreting the robustness of the findings. Another limitation is that detailed clinical and demographic metadata, such as age, sex, disease stage, tumor grade, and treatment history, were not uniformly reported by the original submitters of the GEO datasets used in this study, which precluded more refined subgroup analyses based on these variables. Future investigations should incorporate larger, well-annotated cohorts and experimental approaches to validate these findings.

## 5 Conclusion

This study identifies key up- and downregulated cancer driver genes and suggests a common gene profile between viral and non-viral pathologies in HCC carcinogenesis. By analyzing the interaction between these genes, we determined the importance of considering multiple risk factors in the development of liver cancer. Further *in vitro* and *in vivo* research is crucial for improving our understanding of the molecular basis of HCC pathogenesis and developing more targeted and efficient preventive and therapeutic strategies.

### 5.1 AI disclosure

Artificial intelligence tools were used solely for language refinement and paraphrasing of specific sections of the manuscript. All scientific content, analysis, and interpretations were conducted by the authors.

## Supporting information

S1 Fig Enrichment analysis of up- and down-regulated DEGSs. Gene Ontology for biological processes (BP), molecular function (MF), and cellular component (CC) of common genes among HBV-HCC, HCV-HCC, and HBV-HCV-HCC groups.
(TIF)

S2 Fig KEGG pathway and biological process (BP) of specific genes in each group (HBV, HCV, and HCC). (TIF)

S3 Fig Protein-protein interaction (PPI) network analysis. **(A)** The Venn diagram identifies similar hub genes among the degree, betweenness, eccentricity, and closeness criteria within the HBV-HCV-HCC group. **(B)** Constructing a network using Gephi for similar hub genes that relies on the four criteria.
(TIF)

S4 Fig Gene expression validation of important genes in the TNMplot database, n = 376 in Normal and n = 806 in Tumor tissue. (TIF)

S1 Table The analyzed data of the microarray data (GSE19665, GSE121248, GSE44074, GSE17856, GSE62232, and GSE45050). (XLSX)

S2 Table LogFC of genes related to pathways involved in HCC progression. (DOCX)
