# Supplementary material for: Commonly expressed key driver genes in the pathogenesis of viral and non-viral liver cancer
Source: PLoS One. 2026 Nov 6;21(11):e0349647. doi: 10.1371/journal.pone.0349647 (PMC13742962; doi:10.1371/journal.pone.0349647)
Supplement: Supplementary file 1 [file pone.0349647.s006.docx]

**S2 Table**

|  | **Gene name** | **LogFC in HBV** | **LogFC in HCV** | **LogFC in HCC** |  | **Gene name** | **LogFC in HBV** | **LogFC in HCV** | **LogFC in HCC** |
| --- | --- | --- | --- | --- | --- | --- | --- | --- | --- |
| **Cell cycle and p53 pathway** | *CCNB1* | 2.14 | 0.9 | 2.88 | **PI3-AKT pathway** | *THBS4* | 1.10 | 0.62 | 0.76 |
|  | *MCM3* | 1.07 | 0.78 | 1.99 |  | *ITGB1* | 0.63 | 0.27 | 1.1 |
|  | *SFN* | 2 | 0.57 | 1.3 |  | *FOXO1* | -0.97 | -0.21 | -1.45 |
|  | *CDK1* | 1.81 | 0.22 | 3 | **Wnt pathway** | *GPC3* | 2.68 | 2.95 | 3.42 |
|  | *CDKN2A* | 1.49 | 0.4 | 1.27 |  | *CTNNB1* | 0.6 | 0.2 | 0.65 |
|  | *BUB1* | 1.6 | 0.36 | 2.5 |  | *LEF1* | 1.36 | - | 1.74 |
|  | *TTK* | 2.48 | 0.4 | 2.73 | **The other pathway** | *ADAMTS13* | -1.6 | -0.83 | -1.2 |
|  | *CDC25C* | 1.7 | 0.18 | 1.1 |  | *VWF* | 0.94 | 0.93 | 1.64 |
|  | *PCNA* | 0.9 | 0.17 | 1.46 |  | *SPP1* | 1.7 | 0.5 | 1.32 |
|  | *RBL* | 0.84 | 0.14 | 0.83 |  | *CCL2* | - 1.56 | - 0.51 | -1.38 |
|  | *CDKN2C* | 1.39 | 0.33 | 2 |  | *CCL5* | - 0.94 | - 0.3 | - 0.57 |

*Note:* Not significant for adj-*p*-value, Not significant for Log Fc
